# The Book World of Medicine and Science

**Published:** 1894-10-20

**Authors:** 


### The Book World of Medicine and Science.

Difficult Labour; a Guide to its Management for Students and Practitioners. By G. Ernest Herman, M.D., F.R.C.P. (London: Cassell and Co. 1894; pp. 431.)

Whether as a guide to students and young practitioners, or as a pleasant and readable work of reference to those of more experience, this is an admirable book. While it is not a large and diffuse treatise on the principles of midwifery, it is obvious on every page that it is by reference to principles and by a careful consideration of the causes of the various conditions described that the somewhat dogmatic rules which are laid down for treatment have been arrived at; so that although it is true that the student will find in this book a definite instruction what to do in almost every circumstance in which he is likely to find himself, the guidance given is by no means of a rule of thumb character.

In practice difficult labour is the only condition which the accoucheur has to treat, for, as Dr. Herman says, if the conditions of natural labour are fulfilled "almost the only danger is that of septic infection, and the patient would do quite as well without a doctor as with one." Since, however, we cannot foretell whether in any case these conditions will be fulfilled, skilled help should be available for all lying-in women, and all the more so since "the mother can be safely delivered in spite of almost every complication which makes labour difficult if the abnormal conditions are recognised early and the proper treatment applied in time."

The necessity for practising abdominal palpation is strongly insisted upon in this book, and it is made clear throughout it that the man who is content to get access to the process of parturition only through the vagina, puts himself and his patient under the greatest possible disadvantages both as regards diagnosis and treatment. Knowing as we do how often young medical men in their earlier years of practice accept low-class midwifery largely with the object of gaining experience and manual dexterity, we cannot help feeling that the conditions amid which such cases are attended in the homes of the very poor frequently nullify the very object for which they have been accepted, for every case in which external examination is not pushed to its full limits is a case lost so far as useful experience is concerned.

In the chapter on rupture of the uterus, a good description is given of the changes which take place in the lower segment of the uterus and in the vagina during obstructed labour, and the bearing of these changes on the treatment of the condition is well shown. Considerable stress is also laid on the signs and dangers of tonic contraction of the uterus; in fact, throughout the descriptions of the various forms of obstructed labour, and the dangers which accompany them, the key note as to treatment hinges largely on the occurrence of this state of tonic contraction and the thinning of the vagina and lower segment of the uterus, which so frequently accompanies it.

The exhaustion which occurs in obstructed labour is thus described: "The face becomes anxious; then the pulse gets quicker and smaller; the patient's breathing is hurried in proportion to the pulse. Her tongue becomes first creamy, then yellow, then brown. Her lips get parched. She becomes restless. There may be vomiting. If the patient is not delivered, these symptoms get more and more marked until she dies." These are the well-known symptoms of exhaustion, but it is an exhaustion caused by constant tonic contraction of the uterus, and to temporise means death. "One of the greatest practical blunders that can be made is in the mistaking tonic contraction of the uterus for secondary uterine inertia, otherwise called temporary passiveness, and the reverse. Tonic contraction of the uterus and secondary uterine inertia present certain superficial resemblances. In both regular pains have ceased, in both the patient and her friends may think the labour has lasted too long, and be clamorous for delivery, but yet the diagnosis between them is of extreme importance, for the treatment is diametrically opposite. In tonic contraction of the uterus immediate delivery is absolutely necessary; in secondary uterine inertia it is the worst possible practice, for it ensures post partum hæmorrhage"

Dr. Herman is by no means one of those who would abolish the use of ergot. He points out very clearly the cases in which it may be used and those in which it should never be employed.

The chapter on hæmorrhage after delivery is an admirable survey of the subject, and very clear directions are given as to the various circumstances under which it may occur and the treatment necessary. While many measures are described as means for producing uterine contraction, there is no hesitation, when it is clear that hæmorrhage arises from exhaustion of contractile power, in laying down the law definitely, that the right way is to compress the uterus between one hand in the vagina and the other on the abdomen, and an illustration given showing how this is to be done—the now well known method of bimanual compression.

In view of the discussions which have lately taken place concerning the revived operation of symphysiotomy, Dr. Herman's opinion on the subject is worth quoting: "This operation does not interfere with fertility. Hence it might be required time after time in the same patient. We do not know yet whether, if this operation be repeated several times in the same patient, the parts will heal each time without making the union of the two pubic bones less firm. We know that most patients leave the hospital without lameness or any other apparent ill consequence of the operation; but we do not yet know whether, when the patient resumes a laborious life, she remains free from weakness in the joint. Until evidence is before the profession showing that the operation is without remote detrimental effects, and can be repeated on the same subject without ill consequence, I hesitate to recommend its adoption."

The great advantage of this book to a student, or as a pocket book to a junior practitioner, is that not only are its descriptions clear and comprehensible, but that its directions as to treatment in the various emergencies which arise in practice are definite and distinct, and we do not see how higher praise than this can be given to a book which is put forward as a guide for students and practitioners.

